# Cheburator Software for Automatically Calculating Drug Inhibitory Concentrations from *In Vitro* Screening Assays

**DOI:** 10.1371/journal.pone.0106186

**Published:** 2014-09-03

**Authors:** Dmitry Nevozhay

**Affiliations:** 1 Department of Systems Biology, Unit 950, The University of Texas MD Anderson Cancer Center, Houston, Texas, United States of America; 2 Department of Experimental Oncology, Institute of Immunology and Experimental Therapy, Polish Academy of Sciences, Wroclaw, Poland; 3 School of Biomedicine, Far Eastern Federal University, Vladivostok, Russian Federation; Universidade Federal do Rio de Janeiro, Brazil

## Abstract

In the process of new cancer drug development, as the first step of their assessment, their activities are usually studied *in vitro* against a panel of cancer cell lines. The results of these *in vitro* drug screening assays are commonly expressed as inhibitory concentration 50% (IC_50_): the concentration of the tested agent that inhibits the proliferation of the cancer cell population to 50% of the theoretically possible effect (absolute IC_50_) or maximum effect practically achieved by the drug (relative IC_50_). The currently available software for calculating IC_50_ values requires manual data entry, is time consuming, and is prone to calculation errors. Thus, we have developed open source, free, easy-to-use software for performing standardized data evaluations and automatically calculating the IC_50_. This software eliminates the laborious and error-prone manual entry of data, substantially reduces the amount of time spent for data analysis. It has been extensively used in our department as the main tool for *in vitro* data processing during the past several years and can be useful for other research groups working in the area of anticancer drug discovery, either alone or combined with other software packages. The current version of our program, Cheburator, together with sample data, source code, and documentation, is freely available at the following URL: http://www.cheburator.nevozhay.com (it is free for academic use, but a license is required for commercial use).

## Introduction

High-throughput *in vitro* screening of natural and synthetic compounds is an important first stage in the selection of new anticancer drugs throughout the world. This approach was developed in the late 1980s and evolved from earlier screening programs based on *in vivo* mouse leukemia models. The first large-scale *in vitro* screening program was launched by the U.S. National Cancer Institute (NCI) in 1990 (http://www.dtp.nci.nih.gov). Sixty human cancer cell lines representing 9 types of tumors were selected as the test panel (NCI60 cancer cell line panel). Despite some criticism, the validity of this approach has been demonstrated by the results of almost 2 decades of extensive use [1]. Nowadays, this approach, with various variations has also been adopted in a number of laboratories working in the area of anticancer drug development.

The results of *in vitro* screening tests are often presented as an inhibitory concentration 50% (IC_50_): the concentration of the tested agent that inhibits the proliferation of the cancer cell population to 50% of the theoretically possible effect (absolute IC_50_) or maximum effect practically achieved by the drug (relative IC_50_). The detailed description between the two types of IC_50_ values is provided in the materials and methods section. The data obtained during screening tests and the IC_50_ must be calculated manually, usually using spreadsheet programs. As an alternative, the same calculations can be performed using commercially available statistical software (GraphPad Prism from GraphPad Software) or the drfit package for the R Statistical Environment [2]. However, this approach still requires that data be manually extracted from test files before being processed and entered into the chosen statistical software. No dedicated software package will perform the entire process automatically, including the data extraction and calculation.

We aimed to fill this gap and developed free, open-source, easy-to-use software that was specifically designed to extract data, calculate IC_50_ values, and produce a comprehensive report of the analysis. Our software, Cheburator, is user friendly, customizable, does not require programming or extensive statistical skills, and comes with detailed documentation. It can also process many text-based data files using its built-in convertor. This software has been extensively tested by our research group for several years and has substantially reduced the amount of time spent by our scientific staff on data processing. Because it is free for academic use, it can be successfully adopted by other research groups working in the area of *in vitro* drug screening.

## Materials and Methods

### Experimental design prerequisites for screening *in vitro* tests to obtain good data quality

Cheburator was initially developed to process the data from sulforhodamine B (SRB, [3]) or tetrazolium dye (MTT, [4]) assays, but it can be used with any absorbance or luminescence-based assays. The absorbance values of each cell well in these assays are proportional to the level of the protein (SRB assay) or mitochondrial activity of live cells (MTT assay) in the well. Therefore, these tests are routinely used to evaluate the proliferation of cell populations.

**Figure 1:**
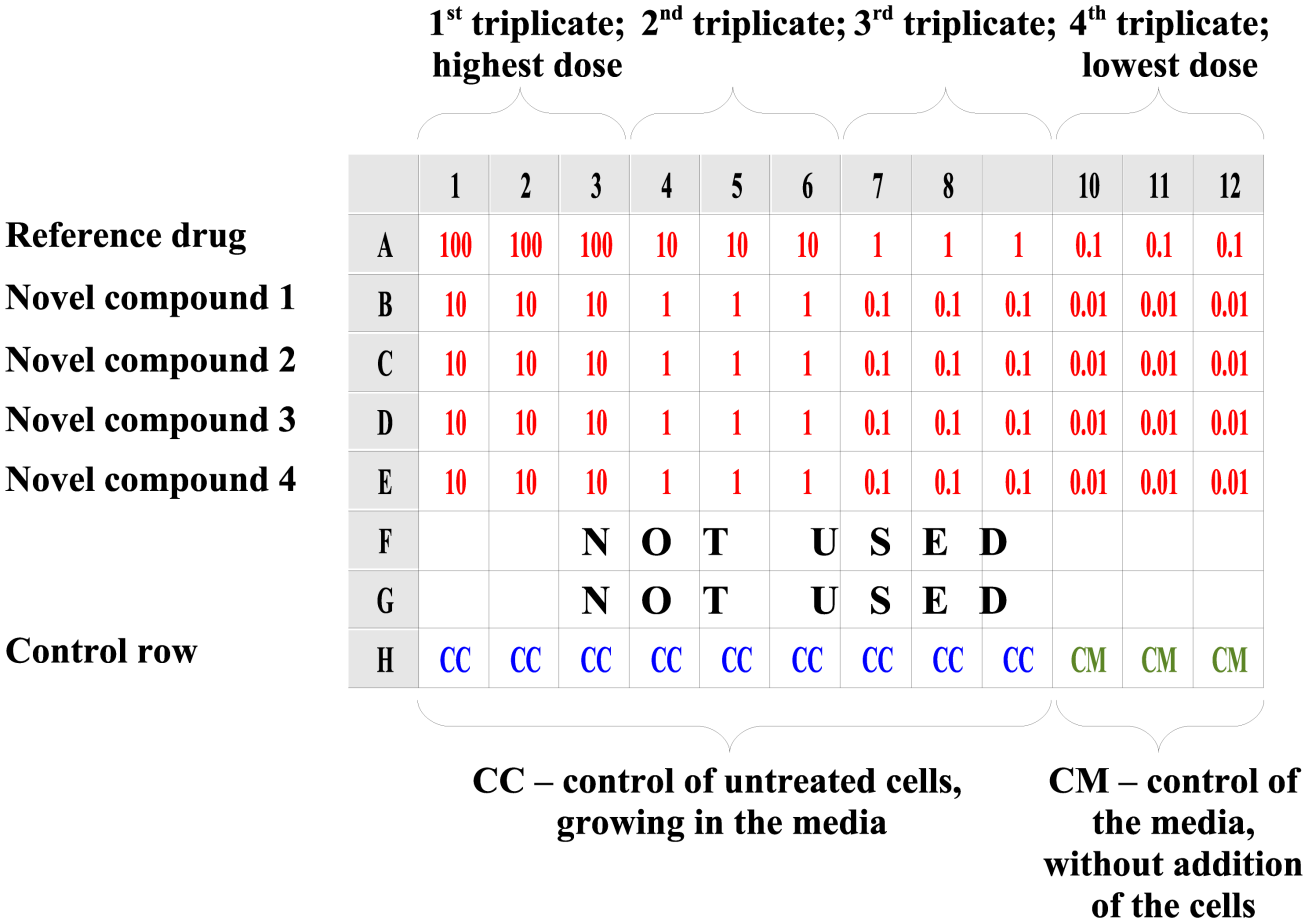
Example test plate designed to be processed in Cheburator. The sample plate with 1 control row (H), 1 row for the reference drug (A), and 4 rows for 4 novel studied compounds (B, C, D, and E, respectively) tested in 4 concentrations. Please note that not all rows have to be used on the plate, and different ranges of concentrations for different compounds can be applied in the test. Concentrations are highlighted in red. Control of untreated cells (CC) and control of media (CM) are in blue and green, respectively.

This software was designed to analyze the results of assays performed on 96-well plates using the layout shown in Figure 1. To ensure good data quality and to minimize impact of pipetting errors, each particular compound concentration is assessed based on the absorbance values from 3 separate wells (triplicate). One row on each plate (usually the last) must contain appropriate control values: the first 9 wells of the row contain untreated cells and the last 3 wells of a row represent background values for the cell culture medium, without cells. The other rows represent data for the compounds of interest, tested in 4 concentrations at 3 wells per concentration (4 concentrations x 3 wells = total 12 wells in each row and compound used in the test). Concentrations have to be arranged from left to right, greatest to smallest.

### Absolute and relative IC_50_ values

**Figure 2:**
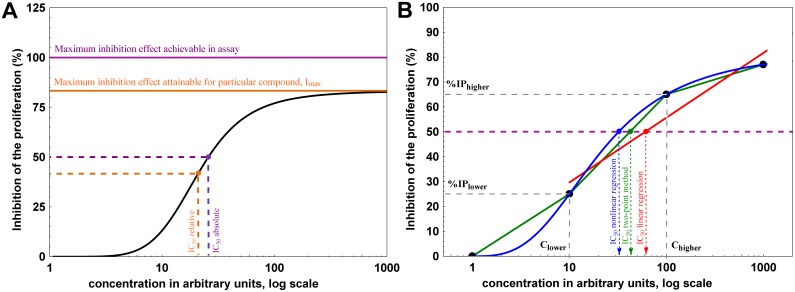
Graphical explanation of data analysis algorithms used in Cheburator. (**A**) Two types of IC_50_ which can be estimated by the software. Absolute IC_50_ is defined as the concentration of tested compound which results in 50% inhibition of cell growth as defined by assay controls (pink lines). Relative IC_50_ is defined as the concentration of tested compound which results in half of the maximum inhibition of cell growth attainable for that particular compound (orange lines). I_max_ is defined as upper plateau of the dose-response curve (solid orange line). (**B**) Example of a dose-response curve drawn on the basis of 4 values (black circles). The simple two-point method uses 2 data points bracketing 50% inhibition of proliferation (green lines) to estimate the IC_50_. In the second method, a linear model is built with the intercept and the slope, which are calculated by linear regression analysis using data points which are >0% and <100% (red lines). These parameters are later used to estimate IC_50_. In the third method, the software uses all data points to build nonlinear regression model and estimate IC_50_ (blue lines). Absolute IC_50_ values are shown on this plot, but software can also estimate relative IC_50_ in linear and nonlinear regression analysis.

Depending on the option selected in the program (*Options* tab, please see below), Cheburator can estimate both absolute and relative IC_50_ values [5], [6]. To understand the difference between the two definitions of IC_50_, let us consider a theoretical dose-response curve where concentration of the studied compound is shown on the abscissa axis and percent of inhibition is shown on ordinate axis (**Figure 2A**). The minimum effect is defined as 0% - complete uninhibited growth of the untreated cells without addition of a studied compound. The maximum inhibition effect achievable and measurable in the assay is 100% - complete inhibition of the cell growth (solid pink line in **Figure 2A**) due to the effect of a studied compound. In this case, absolute IC_50_ is defined as a concentration of studied compound which results in exactly 50% of the maximum inhibition effect achievable in assay (dashed pink line in **Figure 2A**). In practice, even the high concentration of very potent cytotoxic compounds rarely result in 100% inhibition of the cell population. Each drug has a maximum attainable inhibition effect which manifests in an upper plateau of the dose-response curve (solid orange line in **Figure 2A**, also called I_max_). Relative IC_50_ is defined as the concentration of studied compound which results in exactly half of the maximum inhibition effect attainable for that particular compound (dashed orange line).

### Algorithm of the IC_50_ calculation

The percentage of inhibition of cell proliferation in each respective well is calculated relative to untreated cells using the formula:
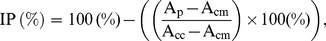
(1)where A_cm_ = mean background absorbance of wells without cells (control of the medium); A_cc_ = mean absorbance in wells containing untreated cells (control of the cells); and A_p = _absorbance value of the well containing cells treated with the tested compound at the concentration of interest.

The percentages of cell proliferation inhibition are used to calculate the IC_50_ value. Cheburator supports three approaches – the simple two-points method previously described by *Reed and Muench* [7], a linear regression, and a nonlinear regression. The default method is nonlinear regression, which is currently considered to be “state-of-the-art” in the field of experimental drug screening [5], [8], [9].

#### Two-points method

This is the simplest among the three methods implemented in our software and can only be used to calculate absolute IC_50_. It assumes linear dose-response relationship in concentrations resulting in near 50% of inhibition and thus, its use is only recommended for preliminary assessment. First, the average percent of inhibition is calculated for each tested concentration (triplicate). Then, the IC_50_ value is estimated by linear interpolation using the 2 data points representing averaged percents of inhibition bracketing 50%. The IC_50_ value or its logarithm, depending on the dose axis transformation, can be graphically represented as the abscissa value for a point of the line drawn between 2 data points where it reaches the value of 50% on the ordinate axis. For example, suppose we have 2 concentrations, 10 and 100 µg/ml, that caused 25% and 65% inhibitions of proliferation, respectively **(** **Figure 2B** **)**. One line could be drawn between these 2 data points (solid green line), and an additional horizontal line could be drawn for the ordinate value 50% (dashed pink line in **Figure 2B**). The abscissa value for the point where these 2 lines intersect is the concentration that causes 50% inhibition in the proliferation of the cell population (green arrow in **Figure 2B**). The program takes into account transformations for the abscissa or ordinate axes if they were applied. For example, when the abscissa is logarithmically transformed and the ordinate axis is not (the default), the IC_50_ value is calculated using the formula:
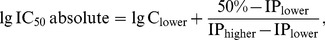
(2)where

C_lower_ = concentration of tested preparation, which resulted in <50% inhibition of proliferation; C_higher_ = concentration of tested preparation, which resulted in >50% inhibition of proliferation; IP_lower_ = inhibition of proliferation (%) calculated for C_lower_; IP_higher_ = inhibition of proliferation (%) calculated for C_higher_.

If one or both data points bracketing the 50% inhibition of proliferation themselves result in 0% or 100% inhibition of proliferation, the IC_50_ value will not be calculated because of the uncertainty of the estimation. It is therefore reasonable to repeat the test for this compound using the narrower dose range, which would provide better data for properly estimating the IC_50_ value. Another case in which the IC_50_ value cannot be properly estimated using the two-points method is when little difference exists between the 2 data points bracketing the 50% inhibition of proliferation. The IC_50_ value would not be estimable if 1 or both data points bracketing the 50% inhibition were in the interval close to 50% (e.g., 48% and 52% by default). Due to the simplicity of the IC_50_ calculation in this method, program does not report 95% confidence intervals for its value in this case.

#### Linear regression method

In this method of calculating the IC_50_ value, the linear regression line is built on the basis of all the data points for which the calculated percent of inhibition is more than 0% and less than 100% (solid red line in **Figure 2B**). The intercept and slope of this line are calculated and used to estimate the IC_50_ value (red arrow and IC_50_ value in **Figure 2B**). However, this method assumes a linear relationship in the entire dose-response curve, which is rarely the case as it typically has a sigmoidal shape [10]. To employ this method for proper IC_50_ calculation, it usually has to be combined with the transformation of 1 or both axes to ensure the proper conversion of the dose-response curve into the linear approximation. The abscissa axis (doses) can be analyzed in either logarithmic scale with the base 2, 5, and 10 (default) or linear scale. The ordinate axis (percent of inhibition), which is linear by default, can also be analyzed using probit, logit, or logarithmic transformations. Goodness of fit in linear regression analysis and therefore the reliability of IC_50_ calculation for each particular compound can be assessed using *r^2^* values reported by the program. If linear regression method is chosen for the IC_50_ value calculation, our program also reports 95% confidence intervals for its value, estimated using the semi-parametric bootstrapping method [11].

#### Nonlinear regression method

This method is currently considered to be “state-of-the-art” for IC_50_ calculation [5], [8], [9] and is used in our software by default. We strongly encourage users of the software to use this method of IC_50_ calculation as it does not require linear relationship between drug dose and percent of inhibition and uses all data points from the test, unless they were specifically discarded from the analysis (see below). The program uses the *HillFit* function from *DMath* library, an open-source mathematical and statistical library developed for Pascal family of programming languages. This function fits logistic model to the experimental data, which is the common way of analyzing sigmoidal dose-response relationships [5] (solid blue line in **Figure 2B**). Because nonlinear regression is essentially an iterative approach, Nelder and Mead method is used for optimizing the parameters and finding the values which correspond best to the experimental data. More technical details on the estimation and optimization algorithms can be found in the *DMath* library documentation and source code, which is also included on the Cheburator home page for convenience. For each tested compound, the program estimates IC_50_ value (blue curve, arrow and IC_50_ value in **Figure 2B**) and I_max_ (a maximum attainable inhibition effect, solid orange line in **Figure 2A**). Similarly to linear regression, the program calculates R^2^ value, which is used to assess goodness of fit. The closer R^2^ value is to 1, the better the model fits the data and the higher the accuracy of IC_50_ estimation. In the case of nonlinear regression analysis, the program also calculates and reports 95% confidence intervals for IC_50_ value, estimated using the semi-parametric bootstrapping method [11].

We recommend that the tests for each compound are repeated several times in independent assays, followed by the calculation of geometric mean of IC_50_ values and its 95% confidence interval. The geometric mean is statistically the most appropriate averaging method for IC_50_ values, due to the fact that they follow log-normal distribution [12].

## Results

### Software implementation and installation

Cheburator was developed using the open-source *Lazarus/FPC 1.0.14* compiler of the Object Pascal programming language for the Windows operating system. We also used *DMath* 0.90 statistical library developed for Pascal family of programming languages. The latest version of the software (1.2.0 at the time of this report) has been tested in Windows XP, Windows Vista, and Windows 7. The source code, including the *DMath* library is freely available for download on software home page (see Software Availability section below) and, in principle, can be used to compile versions running on alternative operating systems using a multi-platform Lazarus/FPC compiler. Cheburator is standalone software; it does not require installation or additional third-party run-time libraries.

### Usage

**Figure 3:**
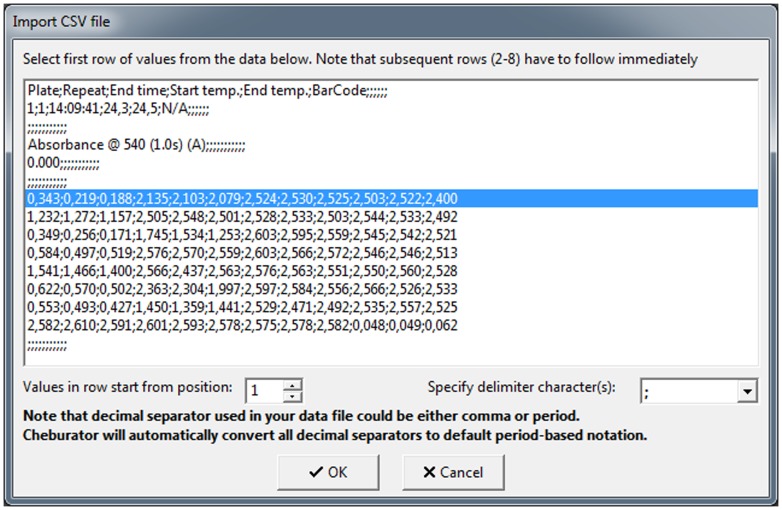
The built-in converter for.csv files. The line containing the data from the first row of the plate is selected in the picture. Other rows have to follow immediately after the first row. All rows before and after the plate data will be discarded. Each row is then divided into separate values using the delimiter specified in the combo box below (semicolon is the default, but other delimiters are also supported). Since each row could contain more values than the number of wells, the user also has to specify in the edit box below which value in the row will be treated as the optical density value for the first well. Data for the subsequent wells have to follow immediately after, separated by delimiters. Note that the user can use a period or comma as the decimal separator for data files. Cheburator will automatically convert all decimal separators into periods, which are the default.

After the software has loaded, users can open a data file containing the results from a single *in vitro* assay. Unless stated otherwise, for the sake of software workflow illustration in this paper, we will use *normal.dat* sample file from the *Samples* folder, which is part of the software package. This file was produced by a Multiskan RC photometer (Labsystems, Helsinki, Finland), which was used in our department at the time of software development. However, to ensure that the software can be used widely, we have added a built-in converter for other data file formats. Presently, the software can import data from text-based, comma-separated files (.csv) that can be exported by Microsoft Excel, LibreOffice Calc, or other commonly used spreadsheet programs (**Figure 3**). The converter is customizable and provides many possibilities for importing other text-based formats, even those of which we are not aware. An example of conversion from a.csv file into Cheburator can be found in the software user manual. In case the built-in converter cannot directly import a data formats into Cheburator, we have included data file format examples with the package that will enable programming additional converters between third-party software and Cheburator if required.

**Figure 4:**
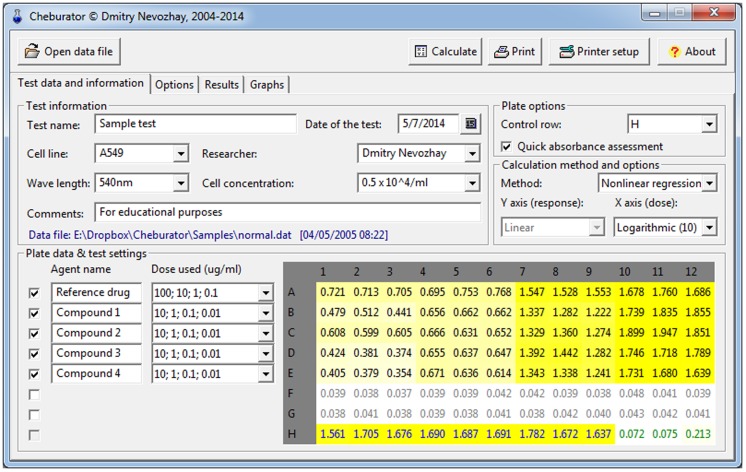
Main window of the program, showing the test parameters and plate data table. The absorbance values in wells are highlighted with the intensities proportional to the ratio between their respective values and control values.

When the data are loaded, the *Test data and information* and *Options* tabs become visible. Users can specify relevant test data, including the name and date of the test, cell line and cell concentration, user name, wavelength used for the assay, and comments in the *Test information* box (**Figure 4**). The path, date, and time of the loaded data file will be shown below and in the final report produced by Cheburator. The absorbance values are shown in the table at the bottom-right part of the window (*Plate data and test setting* box). In the next step, the designated row containing the control values (9 values for untreated cells and 3 values for media) must be selected in the *Control row* combo box (row H in our example) in the right-upper part of the window (**Figure 4**). Cell and medium control values are then displayed in blue and green, respectively. After selection, the optical densities in wells will be highlighted in yellow with the intensities proportional to the ratio between their respective values and control values. The higher the optical density value in a given well, the closer it is to the value of the control of untreated cells and the lower effect observed for the respective drug and the dose used in this well. This absorbance assessment feature is useful for quick visual data checking and comparing the relative potencies of tested chemical compounds. This feature can be turned on and off using the *Quick absorbance assessment* check box located below the *Control row* combo box (**Figure 4**).

Next, scales for the *y* axis (ordinate, inhibition of proliferation in percentages) and *x* axis (abscissa, doses used in the test) are selected using the respective combo boxes in the *Calculation method and options* box. By default, only the abscissa axis is logarithmically transformed; therefore, the concentrations are represented on the decimal log scale, which is common practice in dose-response assays [13]. The final step before analysis is selection of the method used to calculate the IC_50_ (two-point, linear or nonlinear regression, with the latter set by default).

After specifying the relevant details for the test, users select the other plate rows that contain test data for the analyzed compounds using the check boxes located on the left side of the plate data table. The name of the compound and the dose ranges must be specified for every selected row using the *Agent name* edit boxes and *Dose used* combo boxes, respectively. The dose ranges available in the *Dose used* combo boxes are dependent on the type of abscissa axis transformation used (**Figure 4**).

**Figure 5:**
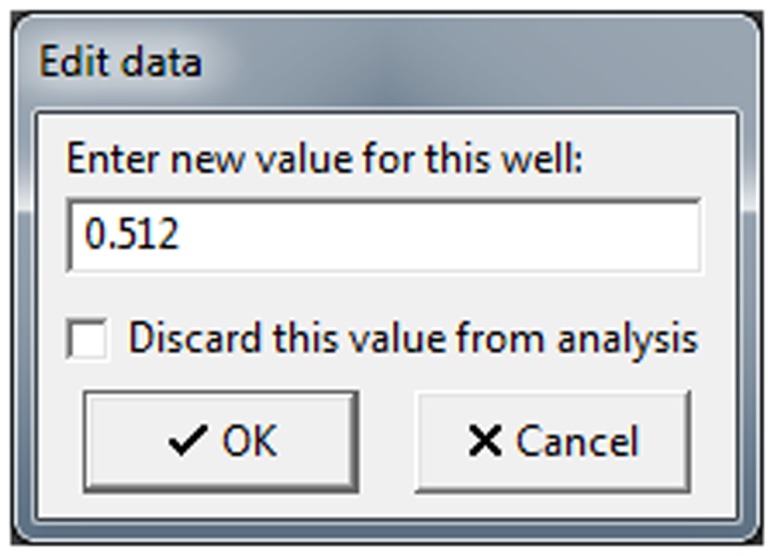
Edit data window. This window allows the user to change or discard particular values from the analysis. Any changes will be noted in the final report.

**Figure 6:**
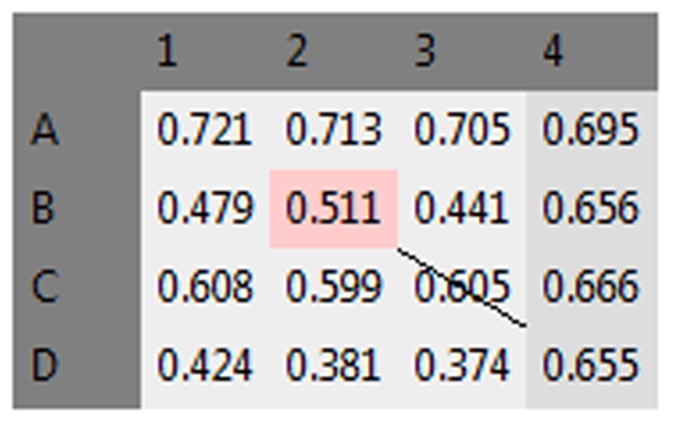
Marking of the discarded and changed values. Changed values appear in red, and discarded values are crossed out after data modification.

It is also possible to discard or modify single values from the plate. To do this, the user must double-click on the desired value in the plate table of the main window (**Figure 4**). The *Edit* data window will be shown (**Figure 5**), and plate values can either be modified or discarded from further analysis using the *Discard this value from analysis* checkbox. Note that the cell will be highlighted in pink or struck out if its value is changed or discarded, respectively (**Figure 6**). Such modified or discarded values will be also marked in the final report printed by the software after analysis.

**Figure 7:**
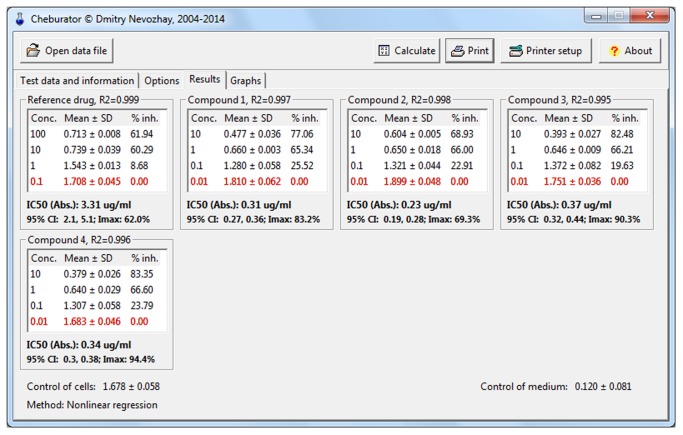
The Results tab after the analysis. It is showing the dose range applied, mean and standard deviations of absorbance values for every triplicate, calculated respective percentages of inhibition of cell proliferation, estimated IC_50_ values, its type, method of calculation, and 95% confidence intervals (for regression analysis).

After the analysis, 2 additional *Results* and *Graphs* tabs will become available (**Figures 7** **and** **8** **,** respectively). The *Results* tab contains the table of results for each compound included in the analysis, including the dose range applied, mean and standard deviations of absorbance values for every triplicate, and calculated respective percentages of inhibition of the cell population. The calculated IC_50_ values (if applicable), their types (absolute or relative), 95% confidence intervals, goodness of fit values (linear and nonlinear regression), I_max_ (nonlinear regression), and the method of estimation are shown below the respective table. Triplicates for which percentages of inhibition of the cell population were calculated to be 0% or 100% are highlighted in red.

**Figure 8:**
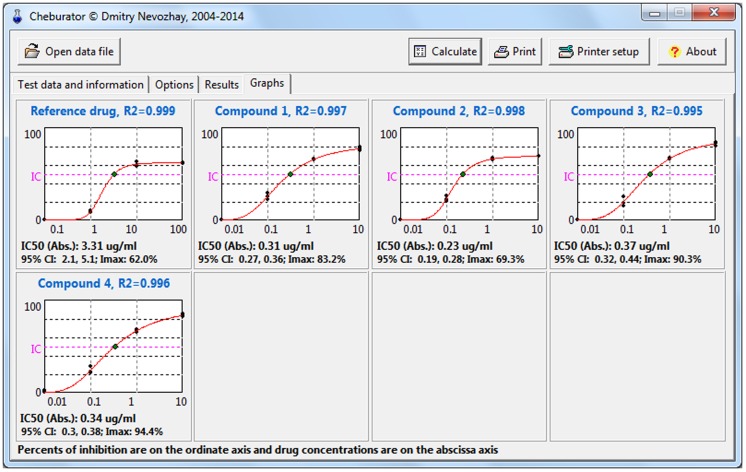
The Graphs tab after the analysis. It is showing the dose-response curves, individual values of percentages of inhibition for each data point, method of calculation used, IC_50_ values, its type, 95% confidence intervals, and goodness of fit (for regression analysis).

User may examine the dose-response curves for each compound analyzed on the *Graphs* tab (**Figure 8**). This option is useful for visually assessing the validity of curves and the method for estimating IC_50_ values. Here, individual data points are plotted in black, which helps to see the experimental variability between absorbance values from different wells. In cases when either the linear or nonlinear regression method is used for estimation, the regression line is also shown in the respective graph. Calculated IC_50_ values (if reached) are shown as green points on the graphs, together with a fuchsia line marking respective percent of inhibition for either absolute or relative IC_50_.

**Figure 9:**
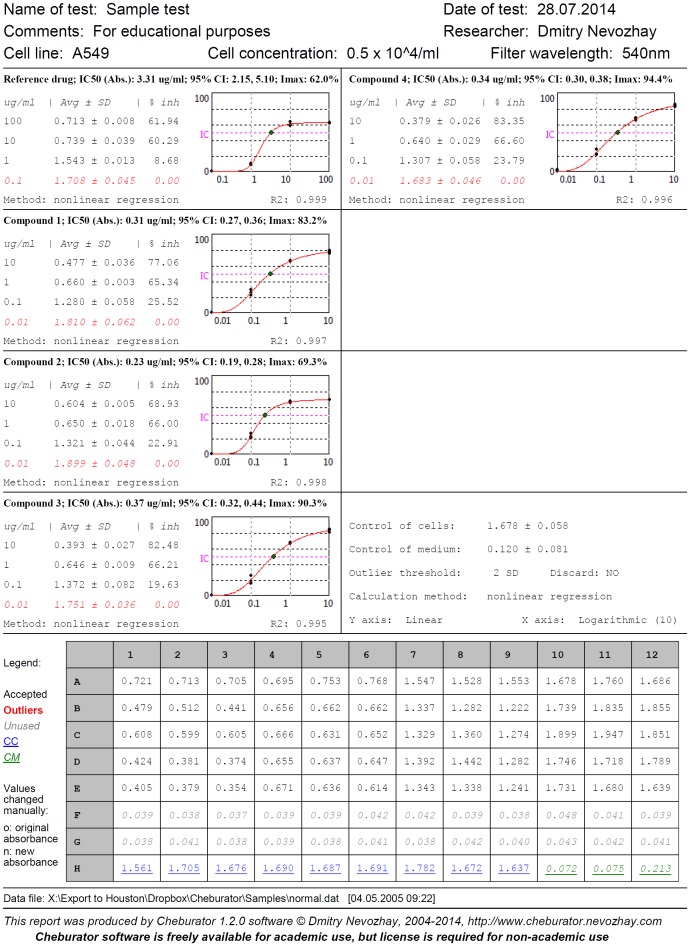
Example of test report printed by Cheburator. This sheet contains the original file’s name, date and time, the name and date of the test, the calculated IC_50_ values, its type, 95% confidence intervals, and goodness of fit (for regression analysis), the percentages of growth inhibition for every concentration and compound, the dose-response curves, the mean optical densities and their standard deviations, the test control values, and the type of the method used for calculation. A table with raw plate data is printed at the bottom part of the report.

Following the analysis, the user can print a detailed report that contains all relevant data: the path to the original file with the raw data, the file’s date and time, the name and date of the test, the calculated IC_50_ values, the percentages of growth inhibition for every concentration and compound, the dose-response curves for visual assessment, the mean optical densities and their standard deviations, the test control values, regression model lines and parameters (if applicable), and the type of the method used for calculation (**Figure 9**). A table with raw plate data is printed at the bottom part of the report. Values are highlighted in color depending on whether they were used in the calculation or were noted as outliers. In case of raw data have been manually modified before analysis, both the original and modified values are printed. In addition, color absorbance highlighting for easy visual data assessment can be printed if the user specifies it on the *Options* tab. Even if an original file is accidentally lost, a hard copy of the report can be used to restore the raw data and repeat the analysis.

**Figure 10:**
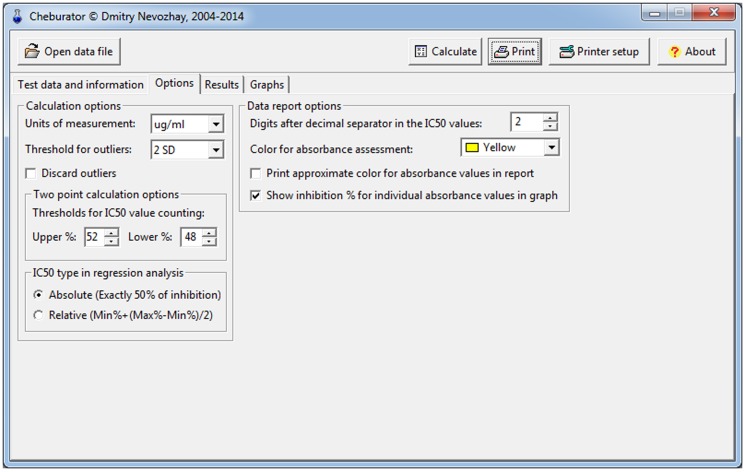
The Options tab. This tab allows user to customize different analysis and report parameters of the software.

Finally, there is also an *Options* tab (**Figure 10**), where users can modify the analysis and report parameters. Turning on the *Discard outliers* checkbox will remove the outliers from the analysis (this option is not recommended and is off by default). Outliers are defined as the values that are outside the mean ± SD for the current triplicate. SD limits can be specified in the *Threshold for outliers* combo box. Below this are the edit boxes in which the upper and lower threshold percentages of inhibition values for two-point IC_50_ calculation method can be specified (52% and 48% by default, as explained in the Materials and Methods section). *IC_50_ type in regression analysis* selection panel allows user to choose whether absolute or relative IC_50_ is calculated during the linear or nonlinear regression analysis. Users can also change the number of digits displayed after the decimal separator in the counted IC_50_ values. In addition, color highlighting of absorbance values can be printed in the final hard copy report if the *Print approximate color for absorbance values in report* checkbox is turned on. The default color for this highlighting can be selected in the combo box above.

### Software Availability

Cheburator is open source and is available for academic use, but a license is required for commercial use. The latest version of Cheburator, together with its source code, sample data files, and documentation, can be downloaded from the program’s web page at http://www.cheburator.nevozhay.com.

## Discussion

Manually estimating IC_50_ values is laborious, time consuming, and prone to calculation errors. Our Cheburator software program has been extensively used in our department as the main tool for *in vitro* data processing during the past several years and has substantially reduced the amount of time spent on these analyses [14]–[18].

Other software exists that can perform IC_50_ calculations, for example, the commercial GraphPad Prism program (GraphPad Software) or the drfit package for R [2]. However, they still require manual data entry or knowledge of R programming. The advantages of our tool are simplicity and convenience for the end user. Cheburator was developed as a specialized software package for processing *in vitro* results; it was designed to minimize the amount of time used for data input and manipulation while simultaneously giving users full control of the analysis process. The program automatically performs calculations that would otherwise require significant time resources, especially with large amounts of data.

If users prefer to fit the final dose-response curves with data in other software packages, our software is still useful for performing reliable preliminary assessments since it requires only minimal data entry and manipulation effort and has features that are useful for visualizing the results. For example, Cheburator could be used during the early screening stages to calculate the intermediate IC_50_ values, assess the approximate magnitudes of drug potencies, or quickly adjust the dose ranges for subsequent tests. We believe that this tool will be useful, either alone or combined with other software packages, for research groups working in the area of anticancer drug discovery.
